# Humoral Immune Response to Inactivated COVID-19 Vaccination at the 3rd Month among People Living with HIV

**DOI:** 10.21203/rs.3.rs-1750225/v1

**Published:** 2022-06-27

**Authors:** Songjie Wu, Shi Zou, Fangzhao Ming, Mengmeng Wu, Wei Guo, Zhongyuan Xing, Zhiyue Zhang, Jinli Liu, Weiming Tang, Ke Liang

**Affiliations:** Zhongnan Hospital of Wuhan University; Zhongnan Hospital of Wuhan University; Wuchang District Center for Disease Control and Prevention; Wuhan University; Wuhan University; Wuhan University; Wuhan University; Wuhan University; The University of North Carolina at Chapel Hill Project-China; Zhongnan Hospital of Wuhan University

**Keywords:** COVID-19, inactivated vaccine, humoral immunity, PLWH, CD4 cell count

## Abstract

**Background:**

Research on the immune response to inactivated COVID-19 vaccination among people living with HIV (PLWH) is limited, especially among those with low CD4+ T lymphocyte (CD4 cell) count. This cross-sectional study aimed to assess the humoral immune response to inactivated COVID-19 vaccination among PLWH compared to HIV negative controls (HNC) and to determine the impact of CD4 cell count on vaccine response among PLWH.

**Methods:**

The neutralizing antibodies (nAbs) and the specific IgM and IgG-binding antibody responses to the inactivated COVID-19 vaccine at the third month after the second dose of inactivated COVID-19 vaccination were measured among 138 PLWH and 35 HNC. Multivariable logistic regression and multiple linear regression models were conducted to identify factors associated with the seroconversion rate of antibodies and the magnitude of anti-SARS-CoV-2 antibody titers, respectively.

**Results:**

At the end of the third month after two doses of vaccination, the seroconversion rates of IgG were comparable between PLWH (8.7%; 95%CI, 3.9–13.5%) and HNC (11.4%; 95%CI, 0.3–22.5%), respectively. The median titers and seroconversion rate of nAbs among PLWH were 0.57 (IQR: 0.30–1.11) log_10_ BAU/mL and 29.0% (95%CI: 21.3–36.8%), respectively, both lower than those in HNC (*P*<0.05). After adjusting for age, sex, comorbidities, and CD4 cell count, the titers and seroconversion rate of nAbs were comparable between PLWH and HNC (P>0.05). Multivariable regression analyses showed that CD4 cell count<200 /μL was independently associated with lower titers and seroconversion rate of nAbs among PLWH (*P*<0.05). A positive correlation was observed between the CD4 cell count and nAbs titers in PLWH (Spearman’sρ=0.25, *P*=0.034).

**Conclusion:**

Our study concluded that the immune response to inactivated COVID-19 vaccination among PLWH was independently associated with CD4 cell count, PLWH with lower CD4 cell count showed a weaker humoral immune response, especially those with CD4 cell count<200 /μL. This finding suggests that expanding COVID-19 vaccination coverage among PLWH is impendency. In addition, aggressive ART should be carried out for PLWH, especially for those with low CD4 cell count, to improve the immune response to vaccines.

## Introduction

Recently, the ongoing pandemic of coronavirus disease 2019 (COVID-19) has posed a serious threat to global public health and economic development [1]. While it is noteworthy that SARS-CoV-2 infection has brought a big challenge to people living with HIV (PLWH). Studies have reported that PLWH had increased risks of more severe disease and deaths from COVID-19[2, 3], possibly as a result of immunosuppression, higher rates of multimorbidity, unsuppressed HIV viral load (HIV-VL), and other determinants of health[4–7].

Nevertheless, limited information is available about the immune response to COVID-19 immunization in PLWH, especially in those with low CD4 + T lymphocyte (CD4 cell) count. Furthermore, the emerging immunogenicity data in PLWH were mostly focused on mRNA COVID-19 vaccines [8–11] or adenovirus vector-based vaccines [12–14], while very few studies focused on the immune response to inactivated COVID-19 vaccines. Two studies that reported the effect of inactivated COVID-19 vaccine on PLWH both had very small sample sizes [15, 16].

WIBP-CorV is an inactivated COVID-19 vaccine. An isolated SARS-CoV-2 strain (WIV-04) was cultivated in Vero cells, chemically inactivated by β-propiolactone, then mixed with an aluminium-based adjuvant[17, 18]. Phase 1 and 2 trials revealed that WIBP-CorV had a low rate of side effects and good immunogenicity[17]. The interim analysis of phase 3 clinical trials showed that the vaccine is 72.8% effective against the symptomatic COVID-19 cases and 100% against severe disease[19]. WIBP-CorV vaccine was one of the most commonly used vaccine in China.

This study aimed to fill this gap by comparing the humoral immune response induced by the inactivated COVID-19 vaccine between PLWH and HIV negative controls (HNC), and determining the impact of CD4 cell count on vaccine response in PLWH.

## Materials And Methods

### Study Participants and Design

The study was conducted from March to October 2021. A total of 138 PLWH and 35 HNC who received two doses of inactivated COVID-19 vaccine (Sinopharm, WIBP-CorV, 4ug/0.5ml, WIV04 strain, Wuhan Institute of Biological Products Co. Ltd) with an interval of 28 days were enrolled in our study. The inclusion criteria for PLWH included the following: (1) age ≥ 18 years old; (2) confirmed HIV infection by HIV-1/2 Western blot assay. Exclusion criteria included the following: (1) presence of severe hearing loss, impaired vision, or intellectual disability observed by the interviewers; or (2) a history of SARS-CoV-2 infection (via serological and nucleic acid test), major psychiatric illness (schizophrenia or bipolar disorder) or neurocognitive impairment; HNC shared the first inclusion criteria and both exclusion criteria with PLWH. Written informed consent was obtained from each participant before screening for eligibility. 138 PLWH and 35 HNC completed immunizations with inactivated COVID-19 vaccine at respective community hospitals and scheduled visits within the prescribed time. Blood samples were collected at baseline (before the first dose vaccination) and the 3rd month after the second dose of COVID-19 vaccination. Data on demographic information, including age, sex, and comorbidities (i.e., hypertension, diabetes mellitus, hyperlipidemia, cancer, chronic cardiovascular and lung, liver, or kidney diseases) were collected from all participants through an electronic questionnaire before vaccination. Clinical and laboratory data regarding the HIV status of PLWH were obtained from the China National HIV/AIDS Comprehensive Response Information Management System (CRIMS). The CD4 cell count of the PLWH and HNC were tested with the blood samples at baseline.

### Immunogenicity Assessments

The primary humoral immunogenicity outcomes included the neutralizing antibodies (nAbs) and the specific IgM and IgG-binding antibody response to the COVID-19 vaccine, measured at baseline and 3rd months after the participants were fully present vacillated with inactivated COVID-19 vaccination. An SARS-CoV-2 nAbs assay kit by surrogate virus neutralization test (Livzon Diagnostics Inc., Zhuhai, China) was used to determine the serum titers of nAbs against the spike protein receptor-binding domain (RBD) according to the manufacturers’ instructions. In brief, SARS-CoV-2 surrogate virus neutralization test detects total immunodominant neutralizing antibodies targeting the viral spike (S) protein receptor-binding domain in an isotype- and species-independent manner. This rapid test is based on antibody-mediated blockade of the interaction between the angiotensin-converting enzyme 2 (ACE2) receptor protein and the receptor-binding domain[17], a positive response is defined as ≥ 10BAU/mL. The semiquantitative of total specific IgM and IgG antibodies were detected using an ELISA kits (Livzon Diagnostics Inc.,Zhuhai, China), which used the recombinant nucleocapsid (N) and RBD antigen of SARS-CoV-2 as coating antigen, following the instruction manual.The qualitative of specific IgM or IgG antibodies was detected using an colloidal gold kit(Livzon Diagnostics Inc.,Zhuhai, China), following the instruction manual.We defined seroconversion of antibodies as a change from baseline seronegative to seropositive.

### Statistical Analysis

Categorical variables were presented as n (%) and compared using the Chi-square test or Fisher’s exact test. Continuous variables with normal distribution were presented as mean (standard deviation [SD]) and compared using t-test or ANOVA analysis, while continuous variables with abnormal distribution were expressed as median (interquartile range [IQR]) and compared using Mann-Whitney U test. Multivariable logistic regression models with 2-sided 95% confidence intervals were conducted to identify factors associated with the seroconversion rate of antibodies. Multiple linear regression was employed to identify factors associated with the magnitude of anti-SARS-CoV-2 antibody titers. Analyses were conducted using SPSS software, version 26.0 (IBM SPSS Inc), and GraphPad Prism 8 for Mac OS X (GraphPad Software, San Diego, CA, USA). A two-sided p < 0.05 was considered statistically significant.

## Results

### Study Participants

Characteristics of the 138 PLWH and 35 HNC were shown in Table 1. PLWH and HNC were similar in age and comorbidities but differed in proportion of male (*P*<0.001). The median (IQR) age of PLWH was 38(31–49) years old, and 88.4% were males. 91.3% of the PLWH were receiving ART and 107(77.5%) had a HIV VL < 50 copies /mL. The CD4 cell count in PLWH was significantly lower than that in HNC [495(IQR: 320–646) vs. 666 (IQR: 534–800) /μL, *P*<0.001].

### Binding-antibody responses to COVID-19 vaccination

At the end of third month after two doses of vaccination, the seroconversion rates of IgM in PLWH and HNC were 3.6% (95%CI: 0.5–6.8%) and 2.9% (95%CI: 0–8.7%), respectively, while no significant difference between the two groups was observed. No significant difference was also found in seroconversion rates of IgG between PLWH (8.7%; 95%CI, 3.9–13.5%) and HNC (11.4%; 95%CI, 0.3–22.5%). After adjusting for age, sex, comorbidities, and CD4 cell count, IgG seroconversion rates were comparable between PLWH and HNC.

### Neutralizing antibody responses to COVID-19 vaccination among PLWH and HNC group

**Figure 1:**
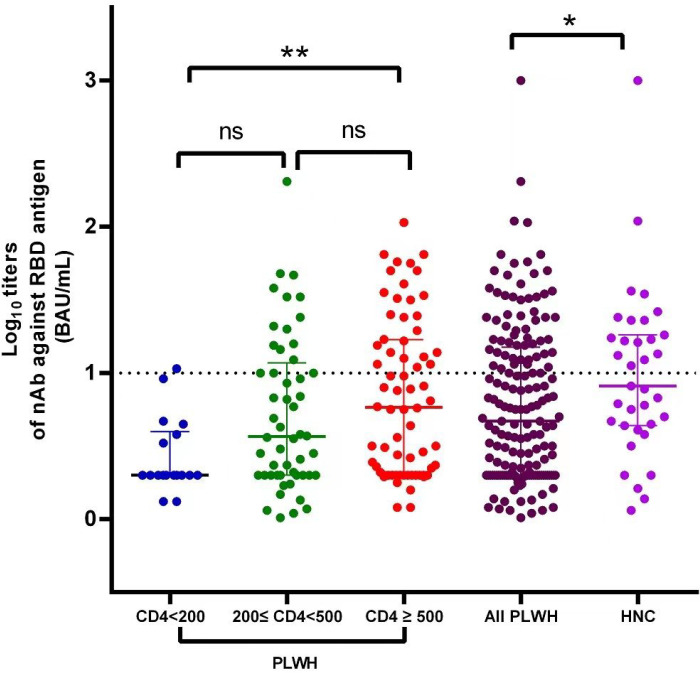
nAbs titers in different groups (PLWH were divided into three groups: CD4 cell count <200/μL, CD4 cell count between 200 and 500 /μL and CD4 cell count ≥ 500/μL). *P<0.05, nAbs titers in PLWH were significantly different from HNC; ** *P*<0.01, nAbs titers in PLWH with CD4 cell count < 200/μL were differ from those in PLWH with CD4 cell count≥500/μL; ns: P>0.05, there were no significantly difference between the groups; *P*-values were computed using the Mann-Whitney U-test.

At the end of third month after two doses of vaccination, the seroconversion rate of nAbs among PLWH was 29.0% (95%CI: 21.3–36.7%), which was significantly lower than that among HNC (48.6%; 95%CI, 31.2–66.0%)]. The nAbs titers among PLWH [0.57 (IQR: 0.30–1.11) log_10_ BAU/mL] was also significantly lower than that among HNC [(median 0.91; IQR, 0.64–1.26) log_10_ BAU/mL](Fig. 1).

In multivariable logistic regression analysis, the people with CD4 cell count<200 /μL tended to have a lower seroconversion rate of nAbs (OR:0.09; 95%CI, 0.01–0.74; *P* = 0.03), as compared to those with CD4 cell count ≥ 500 /μL. Age, sex, comorbidities and HIV infection were not significantly associated with the seroconversion rate of nAbs (Table 2).

We further transformed the nAbs titers (log10) and performed multivariable linear regression analysis. The results determined that nAbs titers in participants with CD4 cell count<200/μL were − 0.21 log_10_ lower than those with CD4 cell count ≥ 500/μL (*P* = 0.012). Age, sex, comorbidities, and HIV infection were not significantly significantly associated with the nAbs titers (Table 3).

### Neutralizing antibody responses to COVID-19 vaccination among PLWH

At the end of third month after two doses of vaccination, the seroconversion rates of nAbs were 5.6% (95%CI: 0–17.3%) in the group with CD4 cell count<200 /μL, 25.0% (95%CI: 12.8–37.2%) in the group with CD4 cell count between 200 and 500 /μL, and 61.8% (95%CI: 49.9–73.6%) in the group with CD4 cell count ≥ 500/μL, respectively. In the multivariable model, participants with CD4 cell count<200 /μL tend to have a lower nAbs seroconversion rate than those with CD4 cell count ≥ 500/μL (*P* = 0.03) (Table 4).

At the end of third month after two doses of vaccination, the median nAbs titers were 0.30 (IQR: 0.30–0.59) log_10_ BAU/mL in the group with CD4 cell count<200 /μL, 0.61 (IQR: 0.30–1.14) log_10_ BAU/mL in the group with CD4 cell count between 200 and 500/μL and 0.81 (IQR: 0.35–1.24) log_10_ BAU/mL in the group with CD4 cell count ≥ 500 /μL, respectively. The nAbs titers were significantly different in three CD4 groups (*P* = 0.009), while participants with lower CD4 cell count<200 /μL tend to have lower nAbs titers (Fig. 1). Multivariable linear regression analysis confirmed this finding(Table 5). There was no significant association between age, sex, comorbidities, HIV-VL, ART, and nAbs titers (*P*>0.05).

**Figure 2:**
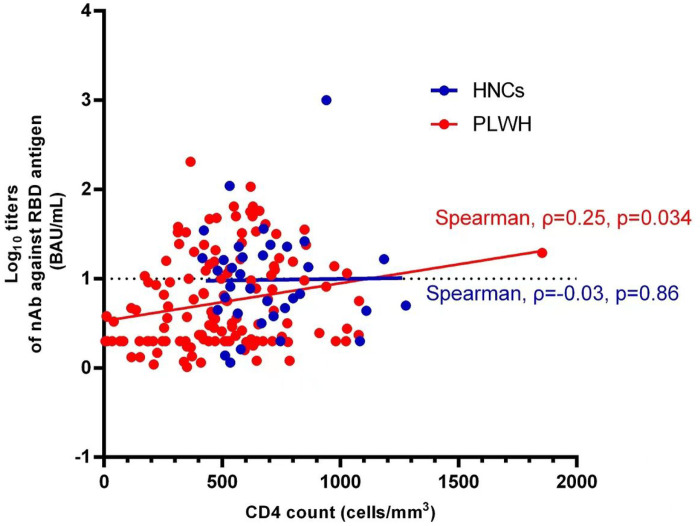
Correlation between CD4 cell count and nAbs titers at the 3rd months after two doses of inactivated COVID-19 vaccination among PLWH and HNC.

The correlation analysis between CD4 cell count and nAbs titers showed a positive correlation in PLWH (Spearman’sρ = 0.25, *P* = 0.034), while no significant correlation between CD4 count and nAbs titers was observed in HNC (Spearman’sρ = 0.03, *P* = 0.86) (Fig. 2).

## Discussion

Understanding the humoral immune response induced by the inactivated COVID-19 vaccine and the impact of CD4 cell count on vaccine response in PLWH were essential in decision-making regarding future disease control and revaccination strategies. It is important to ensure adequate protection against infection in the vulnerable population, especially to prevent the emerging new variants. This cross-sectional study extends the existing literatures [8–16] by providing more comprehensive evidence to assess the inactivated COVID-19 vaccine response among PLWH.

We found that PLWH and HNC had a similar humoral immune response to the inactivated COVID-19 vaccine at the 3rd month after two doses of inactivated COVID-19 vaccination. Even though nAbs titers and seroconversion rate of nAbs in PLWH were both lower than that in the HNC, after adjusting for potential confounders, the differences disappeared. These findings are consistent with the results of other studies conducted in South Africa and UK, which suggested that the immune responses produced by the adenovirus vector-based COVID-19 vaccine among PLWH are similar to those among HNC [12, 13]. Other studies about the immune response to mRNA COVID-19 vaccine among PLWH also reported similar humoral immune response to the healthy controls [8]. The results indicate that PLWH should complete both doses of inactivated COVID-19 vaccine to achieve good protection. Studies have shown that two doses of inactivated CoronaVac vaccines offer high levels of protection against severe disease and death among all age group[21].

Several studies have shown that PLWH have lower responses to some types of vaccine, including hepatitis A, hepatitis B, and influenza vaccine. These responses are dependent on the level of CD4 cell count [22–25]. CD4 cell is pivotal in orchestrating both the humoral and cellular immune responses to vaccination and has an essential impact on antibody production [26]. Some studies also suggested that PLWH with low CD4 cell count had a poor response to the COVID-19 vaccine while PLWH with CD4 cell count in a healthy range mounted equivalent vaccine responses to those in HIV-negative people [27, 28]. Our study found a statistically lower titer and seroconversion rate of nAbs among PLWH with the CD4 cell count<200 μL (versus the group CD4 ≥ 500/μL). We also found a positive correlation between CD4 cell count and nAbs titers in PLWH and CD4 cell count<200/μL independently predicted lower nAbs titers. The results indicate that PLWH, especially those with CD4 cell count<200/μL were still relatively vulnerable even after two doses of inactivated COVID-19 vaccination. A study on the infection forms of SARS-CoV-2 infection among PLWH showed that PLWH were more likely to be an asymptomatic carrier[29]. Prolonged SARS-CoV-2 infection in advanced PLWH with profound immunosuppression or without ART would drive SARS-CoV-2 virus evolution[30], which may be the reason that ‘omicron’ emerged. We should expand COVID-19 vaccination coverage and promote the uptake among the lower- and middle-income countries where the COVID-19 vaccination rates are still low[31], and especially among PLWH. Furthermore, we should strengthen the aggressive ART for PLWH, especially for those with low CD4 cell count, to increase the CD4 cell count and strengthen their immune response level to vaccines and achieve longer duration of vaccines. This is not just to prevent PLWH from SARS-CoV-2 infection but to prevent the emergence of new variants.

This study has several limitations. First, the sample size of HNC was relatively small. Studies with larger sample size will be more conductive to identify individuals who are particularly vulnerable to the impact of SARS-CoV-2 infection and develop targeted vaccination interventions. Second, imbalance existed in the sex distribution of PLWH, which may lead to some bias in our results. However, a previous study found the responses to inactivated COVID-19 vaccination had no significant differences between male and female, which may mitigate some of the sex imbalance in this study [32]. Third, the T-cell responses against the inactivated COVID-19 vaccines weren’t investigated in our study. Long-term follow-up for PLWH with inactivated COVID-19 vaccination will be performed in our further study, and the durability and quality of humoral and cellular responses of inactivated COVID-19 vaccines will be evaluated.

In conclusion, our study indicated that PLWH with lower CD4 cell count showed a weaker humoral immune response to inactivated COVID-19 vaccination, especially those with CD4 cell count<200 /μL. Additional measures against COVID-19 are needed for PLWH who have low CD4 cell count.

## Figures and Tables

**Table 1:** Characteristics of the PLWH group(n = 138) and HNC group (n = 35) in Wuhan, China, 2021

Characteristics	PLWH group(n = 138)	HNC group(n = 35)	Statistics	*P*
Age in years, median (IQR)	38(31–49)	33(29–44)	2.56	0.08
Men, No. (%)	16(88.4)	17(48.6)	28.06	**<0.001**
Comorbidities (%)	17(12.3)	2(5.7)	-	0.26
CD4 cell count /μL, median (IQR)	495(320–646)	666(534–800)	4.12	**<0.001**
CD4 cell count(/μL)				
<200	18	0		
200–500	52	4		
≥500	68	31	18.04	**<0.001**
Receiving ART, n(%)	126(91.3)	-		
ART regimens				
No	12(8.7)	-		
NNRTIs (NVP/EFV)	102(73.9)	-		
INSTIs (EVG/DTG)	14(10.1)	-		
PIs (LPV/r)	10(7.3)	-		
HIV VL <50 copies/mL	107(77.5)			
IgM, n(%)	5(3.6)	1 (2.9)	-*	1.00
IgG, n(%)	12(8.7)	4(11.4)		0.74
nAbs, n(%)	40(29.0)	17(48.6)	4.85	0.03
IgM titers, GMT(95%CI)	0.016(0.014–0.020)	0.018(0.013–0.024)	1.18	0.24
IgG titers, GMT(95%CI)	0.10(0.074–0.13)	0.21(0.14–0.32)	1.97	0.05
Log_10_ nAbs titers, median (IQR)	0.57(0.30–1.11)	0.91(0.64–1.26)	2.46	0.01

Note: NNRTIs: nonnucleoside reverse transcriptase inhibitors, INSTIs: integrase inhibitors, PIs: protein inhibitors, NVP: nevirapine, EFV: efavirenz, EVG: elvitegravir, DTG: dolutegravir, LPV/r: lopinavir/ritonavir

*: no statistics are computed because Fisher exact method was used.

**Table 2:** Factors associated with seroconversion rates of nAbs among PLWH and HNC in Wuhan, China, 2021 (N = 173)

Variables	Adjusted OR(95%CI)	*P*
Age	0.98(0.96–1.01)	0.28
Sex		
Male	Ref.	
Female	2.19(0.87–5.51)	0.10
CD4 cell count(/μL)		
≥ 500	Ref.	
200–500	0.68(0.32–1.45)	0.32
<200	0.89(0.01–0.74)	0.03
Comorbidities		
No	Ref.	
Yes	0.95(0.26–3.46)	0.94
HIV infection		
No	Ref.	
Yes	0.86(0.35–2.15)	0.75

**Table 3:** Factors associated with nAbs titers among PLWH and HNC in Wuhan, China, 2021 (N = 173)

Variables	Estimate	95%CI	*P*
CD4 cell count in 200–500 /μL(versus CD4 ≥ 500/μL)	−0.07	−0.27 to 0.12	0.43
CD4 cell count<200 /μL(versus CD4 ≥ 500/μL)	−0.21	−0.69 to −0.09	0.01
Age	−0.14	−0.01 to 0.001	0.07
Comorbidities (Yes vs No)	0.03	−0.23 to 0.34	0.71
HIV infection (Yes vs No)	−0.07	−0.33 to 0.15	0.45
Sex(Female vs Male)	0.09	−0.11 to 0.36	0.29

**Table 4:** Factors associated with seroconversion rate of nAbs among PLWH in Wuhan, China, 2021 (N = 138)

Variables	Adjusted OR(95%CI)	*P*
Age	0.99(0.96–1.03)	0.62
Sex		
Male	Ref.	
Female	1.57(0.41–6.01)	0.51
CD4 cell count(/μL)		
≥ 500	Ref.	
200–500	0.51(0.21–1.19)	0.12
<200	0.07(0.007–0.77)	0.03
Comorbidities		
No	Ref.	
Yes	0.73(0.14–3.62)	0.70
HIV VL (copies /mL)		
<50	Ref.	
≥ 50	2.51(0.81–7.81)	0.11
ART		
No	Ref.	
Yes	2.40(0.20–28.63)	0.49

**Table 5:** Factors associated with nAbs titers among PLWH in Wuhan, China, 2021 (N = 138)

Variables	Estimate	95%CI	*P*
CD4 cell count in 200–500 /μL (versus CD4 ≥ 500/μL)	−0.11	−0.32 to 0.08	0.24
CD4 cell count<200/μL(versus CD4 ≥ 500/μL)	−0.22	−0.71 to −0.02	0.04
Sex(Female vs Male)	−0.04	−0.38 to 0.23	0.63
Age	−0.14	−0.01 to 0.001	0.11
Comorbidities(Yes vs No)	−0.01	−0.35 to 0.32	0.94
ART(Yes vs No)	0.07	−0.26 to 0.55	0.48
HIV VL (≥ 50 vs <50)	0.16	−0.05 to 0.47	0.11

## Abbreviations

PLWH: People living with HIV
SARS-CoV-2: severe acute respiratory syndrome coronavirus 2
COVID-19: coronavirus disease 2019
HNC: HIV negative controls
nAbs: the neutralizing antibodies
CRIMS: China National HIV/AIDS Comprehensive Response Information Management System
RBD: spike protein receptor-binding domain
ACE2: angiotensin-converting enzyme 2
SD: standard deviatio
IQR: interquartile range
NNRTIs: nonnucleoside reverse transcriptase inhibitors
INSTIs: integrase inhibitors
PIs: protein inhibitors
NVP: nevirapine
EFV: efavirenz
EVG: elvitegravir
DTG: dolutegravir
LPV/r: lopinavir/ritonavir
